# First report on population status and potential distribution of *Tylototritonsparreboomi* Bernardes, Le, Nguyen, Pham, Pham, Nguyen, Rödder, Bonkowski & Ziegler, 2020 (Amphibia, Salamandridae) in Vietnam

**DOI:** 10.3897/BDJ.12.e135451

**Published:** 2024-09-26

**Authors:** Anh Van Pham, Minh Duc Le, Truong Quang Nguyen, Mai Huyen Le, Cuong The Pham, Anh Tuan Nguyen

**Affiliations:** 1 Faculty of Environmental Sciences, University of Science, Vietnam National University, Hanoi, Hanoi, Vietnam Faculty of Environmental Sciences, University of Science, Vietnam National University, Hanoi Hanoi Vietnam; 2 Central Institute for Natural Resources and Environmental Studies, Vietnam National University, Hanoi, Hanoi, Vietnam Central Institute for Natural Resources and Environmental Studies, Vietnam National University, Hanoi Hanoi Vietnam; 3 Department of Herpetology, American Museum of Natural History, Central Park West at 79th Street, New York, USA, Virgin Islands (USA) Department of Herpetology, American Museum of Natural History, Central Park West at 79th Street New York, USA Virgin Islands (USA); 4 Institute of Ecology and Biological Resources, Vietnam Academy of Science and Technology, Hanoi, Vietnam Institute of Ecology and Biological Resources, Vietnam Academy of Science and Technology Hanoi Vietnam; 5 Graduate University of Science and Technology, VAST, Hanoi, Vietnam Graduate University of Science and Technology, VAST Hanoi Vietnam; 6 Faculty of Biology, Vietnam National University, Hanoi, Hanoi, Vietnam Faculty of Biology, Vietnam National University, Hanoi Hanoi Vietnam

**Keywords:** conservation, MaxEnt, new record, Sin Ho District, species distribution modelling

## Abstract

The Sparreboom's Crocodile Newt (*Tylototritonsparreboomi*) is a recently discovered species of crocodile newts from Vietnam and it is currently known only from Sin Ho District in Lai Chau Province. Due to the limited information available on its population status and distribution, the species has been classified as Data Deficient in the IUCN Red List. As a result of our field surveys in 2021 and 2022 in north-western Vietnam, novel data on distribution and population status of and threats to this poorly-known species were collected. We combined the newly-discovered locations with previous occurrence records and used the data as input for modelling the potential distribution of the Sparreboom's Crocodile Newt. The results showed that the Sparreboom's Crocodile Newt’s potential distribution encompasses areas in Lai Chau, Dien Bien and Son La Provinces, Vietnam, as well as a section in Jinping County, Yunnan Province, China. Based on the findings, we suggest that protected areas in the eastern side of Hoang Lien Mountain Range, such as Che Tao, Muong La, Hoang Lien – Van Ban and Bat Xat Nature Reserves and Hoang Lien National Park in Vietnam and Jinping Feishuling Nature Reserve in China be priority sites for the species conservation. Future research and conservation initiatives should prioritise efforts in such areas in an effort to find and protect new populations of the newt.

## Introduction

The Sparreboom’s Crocodile Newt (*Tylototritonsparreboomi* Bernardes, Le, Nguyen, Pham, Pham, Nguyen & Ziegler, 2020) was recently described from the single type locality in Sin Ho District, Lai Chau Province, north-western Vietnam (Bernardes et al. 2020). Its typical habitat consists of secondary forest with medium-sized hardwoods and shrubs (Bernardes et al. 2020). Due to the lack of further in-depth studies, information on its current status, population size, ecology and distribution range of *T.sparreboomi* is still very limited. However, the newt and other related species are already facing severe threats, including deforestation in key habitats, negative impacts from climate change, fragmented distribution range and a high demand from the international pet trade (Bernardes et al. 2021, IUCN SSC Amphibian Specialist Group 2021). As a result, *T.sparreboomi* has been listed in Appendix II of the Convention on International Trade in Endangered Species of Wild Fauna and Flora (CITES) and in the Group IIB of the Governmental Decree No. 84/2021/ND-CP in Vietnam (The Government of Vietnam 2021, Convention on International Trade in Endangered Species of Wild Fauna and Flora 2023).

Species distribution modelling has been widely employed in studying taxonomy, ecology and distribution and developing appropriate conservation measures for many taxa (Phillips et al. 2017, Blair et al. 2022). The approach can provide essential information to identify new sites that may harbour important populations of endangered species (Schingen et al. 2016, Rhoden et al. 2017), to understand evolution processes of highly diverse species complex (Bett et al. 2012, Ngo et al. 2023), to assess the possible impacts of climate change on vulnerable species (Nguyen et al. 2022, Tran et al. 2023b), to recommend protected areas that may be climatically stable for future re-introduction initiatives (Trinh-Dinh et al. 2022) and to find ecologically important regions that can serve as refugia for a number of endemic and threatened taxa in an ever-changing climate (Tang et al. 2018). Several distribution modelling methods have been developed since the early 2000s and amongst the most commonly used is the Maximum Entropy (MaxEnt) method (Elith et al. 2006, Phillips et al. 2006). MaxEnt has been shown to be capable of producing robust results with good predictive performance even for species with a low number of occurrences and it can determine hidden interactions between distribution records and environmental variables (Elith et al. 2006, Pearson et al. 2007, Phillips et al. 2017). Hence, MaxEnt has been recommended as a standard tool for studying species distribution (IUCN Standards and Petitions Committee 2024). A number of studies has successfully employed the method to address ecological and biogeographic issues related to different newt species, from determining the niche diversification patterns in closely-related lineages, investigating habitat preferences in an endemic newt, understanding niche overlap of different groups, to identifying changes in historical distribution of the species (Bernardes et al. 2013, Hernandez et al. 2018, Shu et al. 2022, Tran et al. 2023a).

In this paper, we conducted several field surveys in areas around the type locality of the Sparreboom's Crocodile Newt to collect additional data on distribution and population status of and threats to the species. New data from our field survey were combined with existing localities to create a comprehensive set of occurrence records. We then used this occurrence set as the input for MaxEnt to project the newt potential distribution and recommend conservation measures, based on the modelling results for this threatened endemic amphibian.

## Material and methods

### Sampling

Four field surveys were taken place at 11 sites (within eight communes) in Sa De Phin, Nam Tam, Ta Ngao, Ta Phin, Hong Thu, Phin Ho and Lang Mo communes, Sin Ho District and Nam Ban commune, Nam Nhun District, Lai Chau Province, north-western Vietnam in 2021 and 2022 by A.V. Pham. The study area is characterised by the subtropical climate and seasonal monsoons with annual average rainfall from 2000 to 2100 mm, an annual average air temperature of 23^o^C (minimum to 3.4^o^C and maximum to 39.1^o^C) and relative humidity of 70 to 85% (Bain and Hurley 2011, Tran-Anh et al. 2022, Tran-Anh et al. 2023). The typical habitats at the study sites were undisturbed evergreen forest, disturbed evergreen forest, secondary forest and residential area, with forest cover of approximately 50%. Population densities were calculated on the basis of total individuals per pond with reference to each surveyed transect and total individuals per day (Bernardes et al. 2020, Truong et al. 2020). Newt individuals were observed in water between 09:00 h and 18:00 h in small ponds in evergreen forests. All new occurrence records have been published in the Global Biodiversity Information Facility platform, GBIF as the data package "The Sparreboom's Crocodile Newt's new occurrences in Vietnam" (https://doi.org/10.15468/k4n6x2).

### Species identification

For taxonomic identification, two newt individuals were collected for voucher specimens. After having been photographed in life, animals were anaesthetised and euthanised in a closed vessel with a piece of cotton wool containing ethyl acetate (Simmons 2002), fixed in 85% ethanol and subsequently stored in 70% ethanol. Measurements were taken with a digital calliper to the nearest 0.1 mm. Determination of species was based on morphology following Bernardes et al. (2020). We also sequenced two samples collected from the specimens. The tissue samples were extracted using the protocols of Le et al. (Le et al. 2006). A fragment of the mitochondrial gene, NADH dehydrogenase subunit 2 (ND2), approximately 1100 bp was amplified and sequenced using the primer pair AR SalND2F1 (5’- AAGCTTTTGGGCCCATACC - 3’) (Nishikawa et al. 2014) and TyloR1 (5’- GGTCTTTGGTCTYATTATCCTAA – 3’) (Bernardes et al. 2020). Successful PCR products were sent to FirstBase Malaysia for sequencing. Sequences were compared with those from GenBank using Basic Local Alignment Search Tool (BLAST) searches.

### Species and environmental data pre-processing

To avoid spatial autocorrelation in the distribution dataset, we used the spThin package (Aiello‐Lammens et al. 2015) in R (R Core Team 2024) to thin out localities within 5 km distance (Pearson et al. 2007), resulting in the final set of ten localities. We constructed the MaxEnt models using 19 bioclimatic variables at 30-arc-second resolution available at WorldClim 2.1 database (Fick and Hijmans 2017) and restricted the extent by using a two degree buffer around the minimum convex polygon around occurrence localities (Anderson and Raza 2010). We ran all analyses in Maxent version 3.4.4 (Phillips et al. 2017). However, as Maxent has a tendency to produce overfitting models (Merow et al. 2013), we performed the following set of tuning steps to minimise overfitting and maximise discriminatory ability using ENMeval package (Kass et al. 2021) in R.

### Modelling

We performed the tuning process using all feature class combinations and tested the models with the regularisation multiplier ranging from 1.0 to 10.0 by increments of 0.5. Other model parameters, for example, convergence threshold and feature selection, followed recommendations from model developers (Phillips et al. 2006). We then used the jackknife method, recommended for species with a low number of occurrences (Pearson et al. 2007), to construct the models. To assess model performance and select the most suitable one, we used the 10% omission rate threshold to select models that showed the least overfitting. Of this subset, the models with the highest AUC values were selected. Final models were then compared using the Akaike Information Criterion (AIC), which balances complexity with model fitness (Warren and Seifert 2011). For the final model, we used the 10% training presence threshold to classify between suitable and unsuitable areas for the Sparreboom's Crocodile Newt (Pearson et al. 2007).

## Results

### New records of the Sparreboom's Crocodile Newt in Lai Chau Province

**Figure 1.**
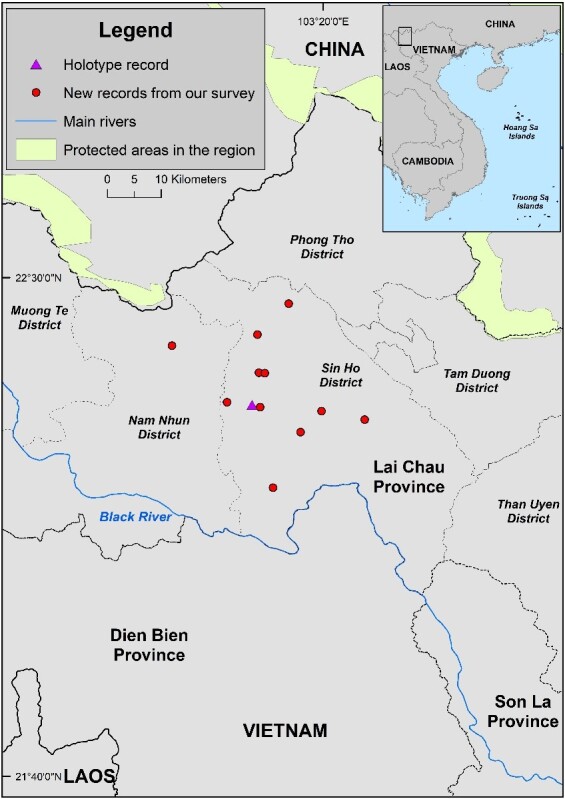
New locality records of the Sparreboom's Crocodile Newt in Lai Chau Province, Vietnam.

The Sparreboom's Crocodile Newt was previously known only from the type locality in Sa De Phin Commune, Sin Ho District in Lai Chau Province (Bernardes et al. 2020). In this study, we discovered additional records of the species in surrounding areas, including Nam Tam, Ta Ngao, Ta Phin, Hong Thu, Phin Ho and Lang Mo communes, Sin Ho District and Nam Ban Commune, Nam Nhun District, Lai Chau Province (Fig. 1). The distance from the new records to the type locality ranged from approximately 1.5 to 20 km. Two 1035 bp long sequences (GenBank accession numbers PQ351754 and PQ351755) obtained from the collected samples were identical and 99.90% similar to that with accession numbers MT210163.1 available on GenBank.

**Figure 2.**
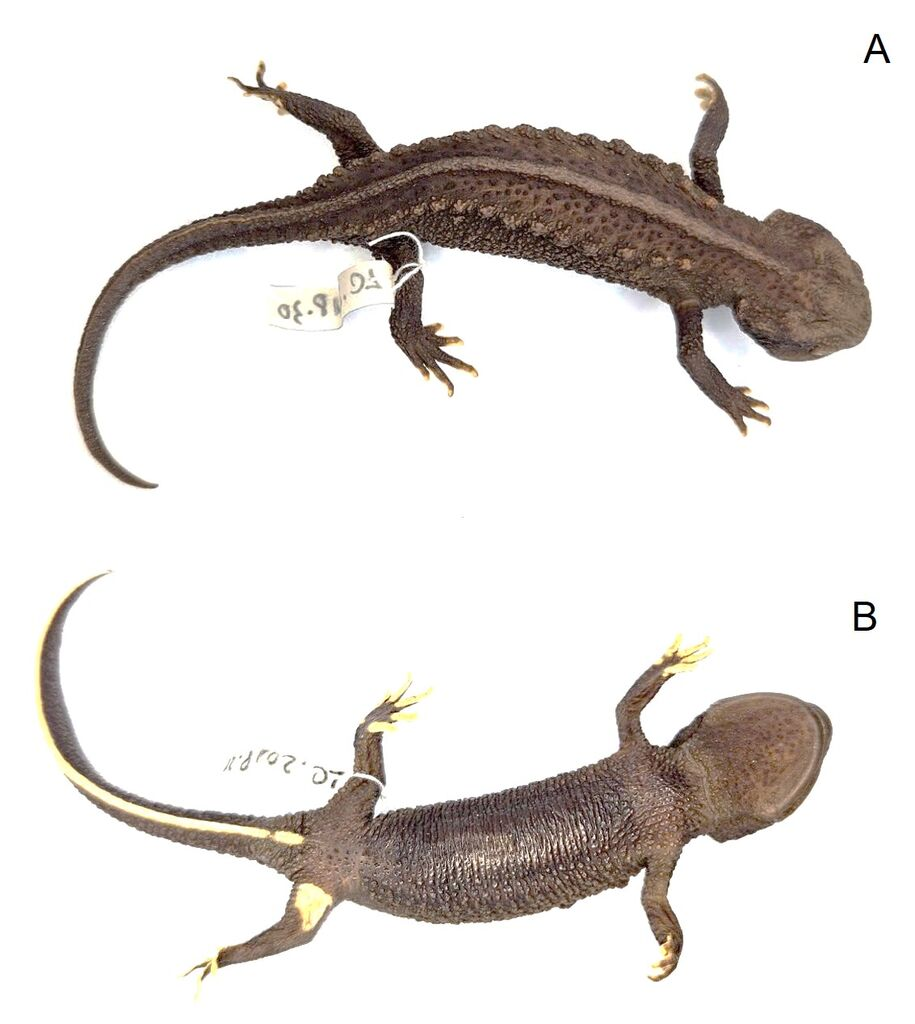
*Tylototritonsparreboomi* in preservative: **(A)** dorsal view; **(B)** ventral view.

Morphological characteristics of specimens collected in Lai Chau Province match well with the diagnosis of *T.sparreboomi* (Bernardes et al. 2020): SVL 73.8 mm in male (n = 1) and 74.0 mm in female (n = 1). Habitus stout; head longer than wide; snout wider than long; nostrils close to snout tip; labial fold slightly evident; dorsolateral bony ridges on head wide, protruding, from above eye to above anterior end of parotoid, posterior ends scrolled inside; mid-dorsal ridge on head indistinct; parotoids enlarged, projecting backwards; glandular vertebral ridge high, wide, smooth and segmented extending from top of head to base of tail, separated from mid-dorsal ridge; tips of fore- and hind limbs overlap when adpressed along body; tips of fingers reaching nostril when fore-leg laid forward; and tail laterally compressed, thin and tip acuminated (Fig. 2).

### Population status of the Sparreboom's Crocodile Newt

**Figure 3.**
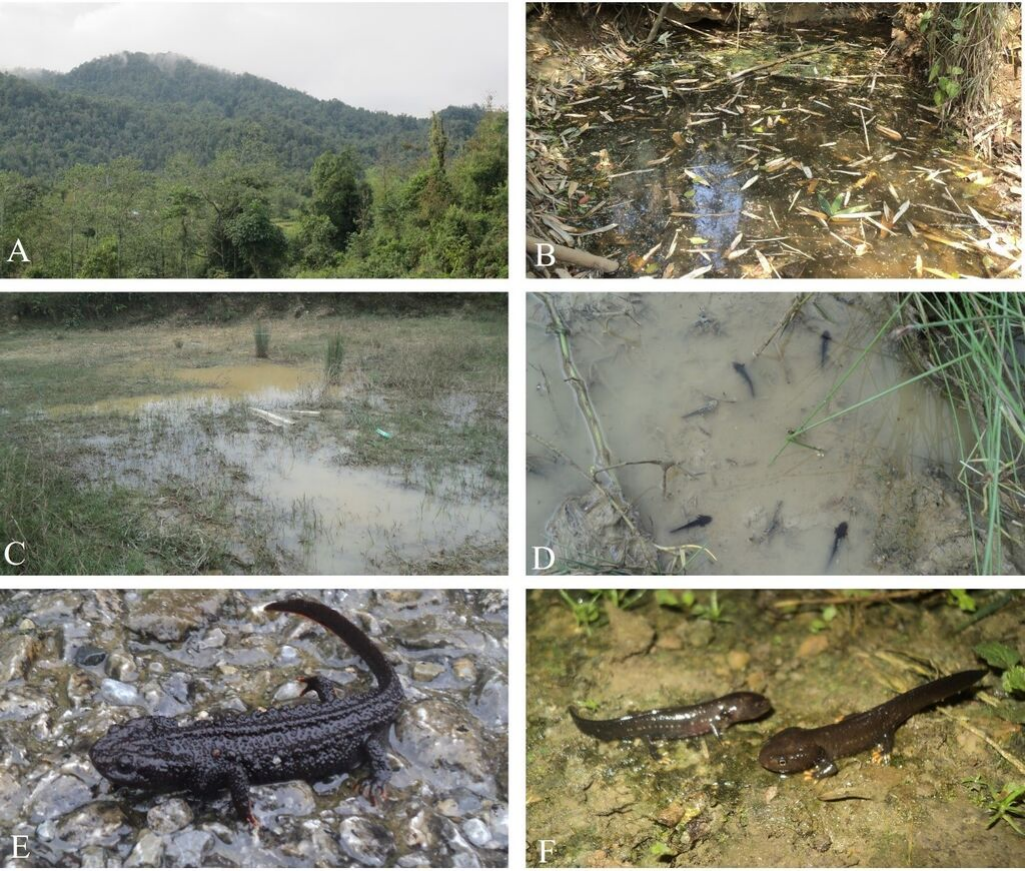
*Tylototritonsparreboomi* recorded in Sin Ho District, Lai Chau Province, Vietnam: **(A)** Evergreen forest habitat in Sa De Phin Commune; *(B)* Pond 1 in Sa De Phin Commune; **(C)** Pond 2 in Sa De Phin Commune; **(D)** Larvae in pond 2; **(E)** Adult male; and **(F)** Two juveniles. Photos by A.V. Pham.

During the field research, *T.sparreboomi* was encountered in 12 ponds at 11 sites in seven communes of Sin Ho District and one commune of Nam Nhun District, Lai Chau Province (Fig. 1). A total of 35 individuals (14 adult males, 19 adult females and two juveniles) and many larvae were observed (Fig. 3, Table 1). We observed from one to five individuals per pond. Remarkably, we found many larvae in two ponds that were about to dry up (Fig. 3). Adults and juveniles were detected during the rainy season (from April to July), while larvae were seen in October. Thus, it may be suggested that the breeding season of this species takes place in the rainy season, from April to July.

### Ecological notes

The Sparreboom's Crocodile Newt individuals were visible mostly during the daytime, between 9:00 h and 18:00 h. The surrounding habitat was the mixed evergreen forest of medium hardwoods, shrubs and arrowroot. They were found at elevations between 860- and 1.550 m. The relative humidity was approximately 70–85% and the air temperature ranged from 22^o^C to 28^o^C. The ponds were approximately 2–15 m in width, 5–25 m in length and 0.1–1.5 m in depth (Fig. 3).

### Potential distribution of the Sparreboom's Crocodile Newt

**Figure 4.**
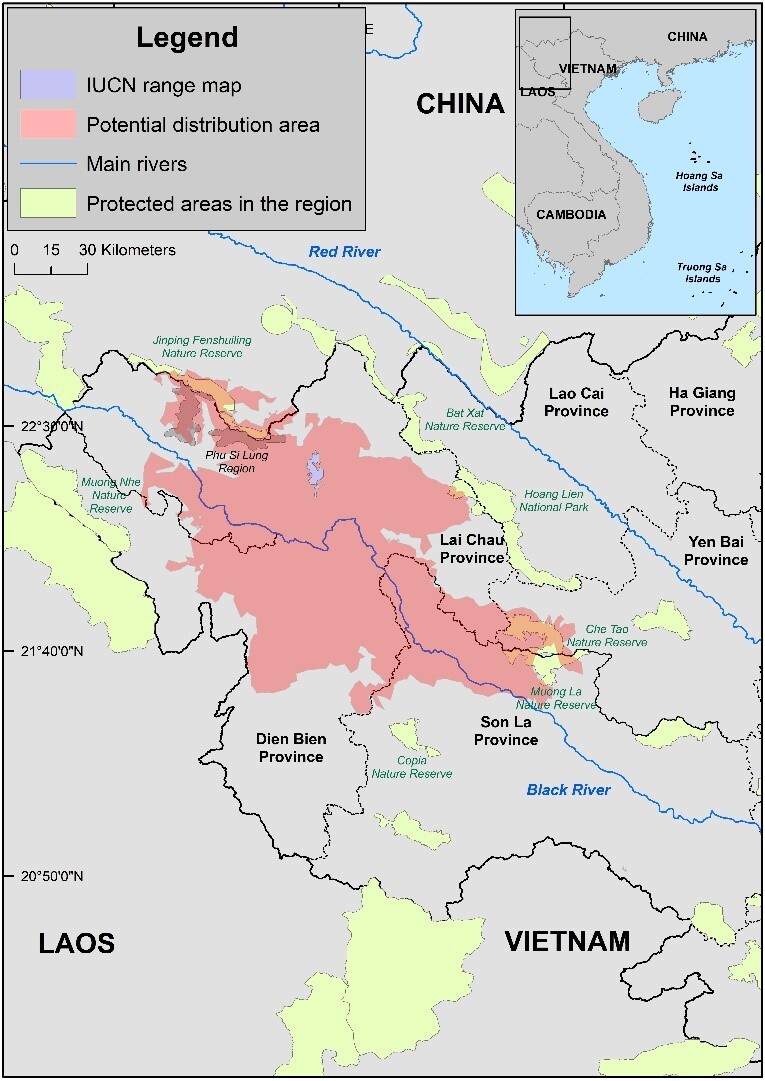
Potential distribution range of the Sparreboom's Crocodile Newt.

For the modelling results, Maxent models showed reasonable prediction power for the distribution of the Sparreboom's Crocodile Newt, with the average AUC values > 0.94. The optimal model had the regularisation multiplier value of 2.0 and a combination of linear and quadratic feature classes and the AUC value of 0.9478. All final models were quite similar in terms of predicting the overall distribution of the species and only differed slightly in exact locations and total suitable areas (Fig. 4). However, the regularisation multiplier value of 2.0 for the optimal model means that the final result was a bit restricted and might infer a smaller distribution area than the actual range of the species. Therefore, the final prediction should be cautiously interpreted as “high potential zones” and it may exclude regions that are less suitable climatically, but are still good for the Sparreboom's Crocodile Newt, especially regions near the “edge” of the predicted distribution range. The current potential distribution range of the Sparreboom's Crocodile Newt is estimated at about 12,900 km^2^. In particular, the suitable area covers a large continuous part in Lai Chau, Dien Bien and Son La Provinces of Vietnam and a small part in Jinping County, Yunnan Province of China. Compared to the IUCN range map (IUCN SSC Amphibian Specialist Group 2021), our results suggest a significantly larger distribution area for the newt, including more locations in the ‘edge’ areas of the range, such as Dien Bien and Son La Provinces. However, our projection is based solely on bioclimatic variables, which does not take into account other potential interactions, such as natural barriers, species interactions or human impacts and, therefore, our results should be viewed with caution.

## Discussion

The modelling results estimate that the current potential distribution range of the Sparreboom's Crocodile Newt covers nearly 13,000 km^2^ with a large continuous part in Lai Chau, Dien Bien and Son La Provinces, Vietnam and a small part in Jinping County, Yunnan Province, China. A number of protected areas in the region may harbour the species populations and future studies may focus on Muong La Nature Reserve (Son La Province, Vietnam) and Che Tao Nature Reserve (Yen Bai Province, Vietnam). While located in different provinces, the protected areas form a continuous forest block covering about 38,000 hectares and were established to secure the remaining populations of the Western Black-crested Gibbon (*Nomascusconcolor*) in Vietnam. There has been little effort to document the herpetofaunas in the nature reserves. However, recent studies have unveiled several new amphibian taxa in the area (Pham et al. 2019, Pham et al. 2019a, Le et al. 2021, Pham et al. 2023). Hence, further field surveys should be prioritised to find additional potential populations of the Sparreboom's Crocodile Newt there.

Hoang Lien National Park (Lai Chau and Lao Cai Provinces, Vietnam), Hoang Lien – Van Ban Nature Reserve (Lao Cai, Vietnam) and Bat Xat Nature Reserve (Lao Cai Province, Vietnam) together establish a contiguous protected landscape of around 74,000 hectares, which is home to many endemic amphibians (Bain et al. 2006, Rowley et al. 2013, Tapley et al. 2020). While Hoang Lien Range itself may serve as a dispersal barrier for many reptiles and amphibians (Bain and Hurley 2011), there have been records of amphibians that inhabit both Hoang Lien and surrounding lowland regions (Ohler et al. 2000, Zhang et al. 2010, Pham et al. 2019b, Luong et al. 2021). Future surveys should be conducted in Hoang Lien region to confirm the presence of the newt. Based on our modelling results, it is also clear that establishing a biological corridor that connects two main areas, including Muong La – Che Tao and Bat Xat – Hoang Lien – Van Ban, could be highly beneficial for long term conservation of many endangered and endemic reptiles and amphibians, including the *T.sparreboomi*, in north-eastern Vietnam.

Jinping Feishuling Nature Reserve (Yunnan, China) together with Phu Si Lung Region (Lai Chau, Vietnam) is a transboundary forested region of about 78,000 hectares, which harbours a number of amphibian species, but the forested area on the Vietnam’s side is not yet protected (Yu et al. 2008, Zhang et al. 2010, Li et al. 2012). Setting up a new protected area in Phu Si Lung would be important for protecting rare and threatened amphibians in the area.

Our results also show that the distribution of the Sparreboom's Crocodile Newt does not extend past the Hoang Lien Mountain Range. In the East of Hoang Lien Range is the Red River and it has been proposed that either the Red River alone or the combination of both Hoang Lien Mountain Range and the Red River can act as a natural dispersal barrier for many species of the north-eastern herpetofauna (Bain and Hurley 2011). Our models provide further evidence for such hypothesis with a significant bioclimatic effect created by the Hoang Lien Mountain Range.

**Figure 5.**
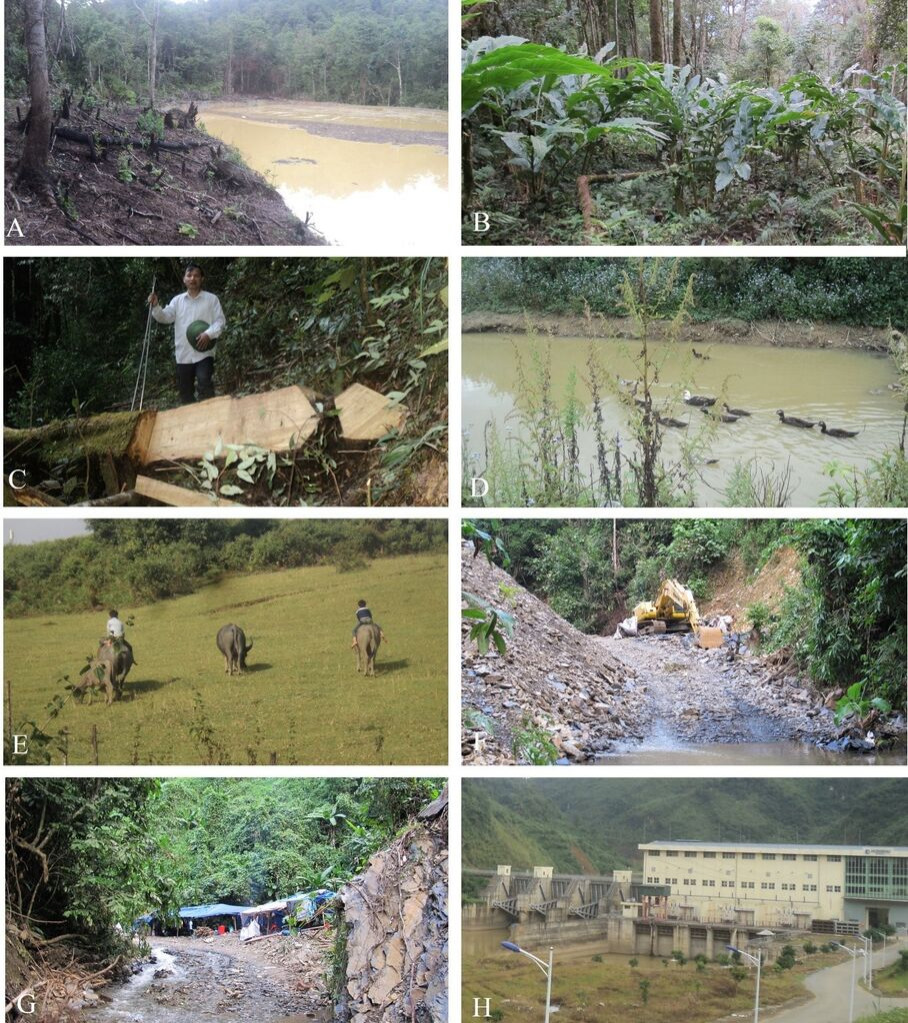
Threats to the habitat of *Tylototritonsparreboomi* in Lai Chau Province, Vietnam: **A** Farming in the evergreen forest in Sa De Phin Commune; **B** Plantation of cardamom in evergreen forests in Sa De Phin Commune; **C** Illegal timber logging in Ta Phin Commune; **D** Poultry raising in the forest in Sa De Phin Commune; **E** Cattle raising near the forest in Sa De Phin Commune; **F, G** Gold mining activities in Ta Phin Commune; and (H) Hydropower dam construction in Ta Phin Commune. Photos by A.V. Pham.

Based on our field surveys, major threats to the Sparreboom's Crocodile Newt in Lai Chau Province include habitat loss and degradation. Deforestation resulting from agricultural activities was observed in April 2022 in Sa De Phin Commune, Sin Ho District (Fig. 5A and B). Illegal timber logging was evident in the forest of Ta Phin Commune, Sin Ho District Province (Fig. 5C). Cattle and poultry farming was also recorded in Sa De Phin Commune, Sin Ho District (Fig. 5D and E), causing environmental pollution as well as other disturbances. Gold mining was also seen in Ta Phin Commune, Sin Ho District (Fig. 5F and G). In addition, we witnessed a small hydroelectric dam in Ta Phin Commune (Fig. 5H), which disrupted the natural habitat of *T.sparreboomi* by fragmenting intact forests and altering water-flowing streams. As the species has only been found in forested habitat, it is likely highly vulnerable to deforestation, which has been occurring throughout the region (IUCN SSC Amphibian Specialist Group 2021).

## Figures and Tables

**Table 1.** New records of the Sparreboom's Crocodile Newt in Lai Chau Province, Vietnam.

**Location**	**Microhabitat**	**Observed Individual**
1. Sa De Phin Commune, Sin Ho District	Pond 1: The pond is approximately 4.5 m in width, 6 m in length and 0.8 m in depth near the edge of the evergreen forest with medium hardwoods, shrubs and cardamom.	Three adult males, one adult female, two juveniles and many larvae were observed between the hours of 15:00 h and 17:00 h.
	Pond 2: The pond is approximately 2 m in width, 5.5 m in length and 0.5 m in depth near the edge of the evergreen forest with medium hardwoods, shrubs and cardamom.	One adult male, two adult females and many larvae were observed between the hours of 10:00 h and 16:00 h.
2. Sa De Phin Commune, Sin Ho District	Pond 3: The pond is approximately 15 m in width, 25 m in length and 1.5 m in depth near the edge of the evergreen forest with medium hardwoods, banana, bamboo, cardamom and shrubs.	Two adult males and three adult females were observed between the hours of 09:00 h and 11:00 h.
3. Ta Phin Commune, Sin Ho District	Pond 4: The pond is approximately 2 m in width, 3 m in length and 0.1 m in depth in evergreen forest with medium hardwoods, cardamom and shrubs.	One adult female was observed at 14:05 h.
4. Nam Tam Commune, Sin Ho District	Pond 5: The pond is approximately 6 m in width, 10 m in length and 0.5 m in depth in evergreen forest with medium hardwoods, banana and shrubs.	Two adult females were observed between the hours of 16:00 h and 17:00 h.
5. Nam Ban Commune, Nam Nhun District	Pond 6: The pond is approximately 2.5 m in width, 5 m in length and 0.5 m in depth in evergreen forest with medium hardwoods, bamboo and shrubs.	Three adult males and two adult females were observed between the hours of 16:00 h and 17:30 h.
6. Hong Thu Commune, Sin Ho District	Pond 7: The pond is approximately 2 m in width, 6 m in length and 0.3 m in depth in evergreen forest with medium hardwoods, bamboo, banana and shrubs.	Two adult males and two adult females were observed between the hours of 15:30 h and 17:00 h.
7. Phin Ho Commune, Sin Ho District	Pond 8: The pond is approximately 2 m in width, 3 m in length and 0.1 m in depth near Road at the edge of the evergreen forest with medium hardwoods, cardamom and shrubs.	One adult male was observed at 14:30 h.
8. Lang Mo Commune, Sin Ho District	Pond 9: The pond is approximately 3 m in width, 5.5 m in length and 0.3 m in depth in evergreen forest with medium hardwoods, bamboo and shrubs.	One adult male and two adult females were observed between the hours of 16:30 h and 17:00 h.
9. Ta Phin Commune, Sin Ho District	Pond 10: The pond is approximately 2.5 m in width, 4 m in length and 0.5 m in depth in evergreen forest with medium hardwoods, bamboo, cardamom and shrubs.	Two adult females were observed between the hours of 16:00 h and 17:00 h.
10. Lang Mo Commune, Sin Ho District	Pond 11: The pond is approximately 4 m in width, 5 m in length and 1.0 m in depth in evergreen forest with medium hardwoods and shrubs.	One adult male was observed at 16:20 h.
11. Ta Ngao Commune, Sin Ho District	Pond 12: The pond is approximately 2.5 m in width, 5.5 m in length and 0.8 m in depth in evergreen forest with medium hardwoods, bamboo, banana and shrubs.	Two females were observed at 17:30 h.
